# Targeting DNA repair pathways to overcome cancer drug resistance

**DOI:** 10.20517/cdr.2021.80

**Published:** 2021-08-19

**Authors:** Robert C.A.M. van Waardenburg, Eddy S. Yang

**Affiliations:** 1 Department of Pharmacology and Toxicology, University of Alabama at Birmingham, Birmingham, AL 35233, USA.; 2 Department of Radiation Oncology, University of Alabama at Birmingham, Birmingham, AL 35233, USA.; 3 O’Neal Comprehensive Cancer Center, University of Alabama at Birmingham, Birmingham, AL 35233, USA.

DNA damage response and DNA repair pathways are evolutionarily conserved from prokaryotes to eukaryotes to protect the host from genomic instability. Dysregulation of proteins involved in these pathways in mammalian cells increases genomic alterations leading to genomic instability, a well-established hallmark of cancer^[1,2]^. However, our understanding of the signaling pathways to repair DNA damage in cancers has grown exponentially over the last decades. Because one of the mainstays of successful cancer treatments acts through generating DNA damage, the growth of understanding these pathways have led to emerging and promising strategies of targeting the DNA damage response and DNA repair pathways to enhance cancer cell sensitivity to current chemotherapeutic agents. Additionally, understanding this biology can also improve the therapeutic index, which emphasizes the effective killing of cancer cells while sparing healthy cells in the patient. Moreover, by targeting signaling and repair of DNA lesions, we aim to prevent compensatory activation of DNA repair pathways as a resistance mechanism. As such, the concept of targeting DNA repair has been successfully developed in the last decade to a bona fide therapeutic strategy with poly (ADP-ribose) polymerase inhibitors (PARPi) to treat DNA repair deficient breast, ovarian, pancreatic, and prostate cancers^[3-9]^. These successes have stimulated the clinical development of small molecules that target other key components of the DNA damage response and repair pathways. These include DNA-dependent protein kinase (DNA-PK), ATM and Rad3 related kinase (ATR), ATM and checkpoint kinase 1 (CHK1). Furthermore, in the era of precision medicine, the precise targeting of these key players provides an opportunity to utilize biomarkers of DNA repair defects to select the optimal treatment for each patient in order to maximize the therapeutic index.

In this Special Issue of *Cancer Drug Resistance*, we present a collection of basic and translational articles discussing the roles that the DNA damage response and repair pathways play in cancer response to treatment as well as resistance.

In the first article, Nickoloff *et al.*^[10]^ discuss exploiting the DNA repair pathways to improve radiosensitivity of the tumor and to prevent resistance while also protecting healthy tissue during radiotherapy. Currently, more than half of the cancer patients are treated with radiotherapy, which induces among others cytotoxic DNA lesions. This review highlights the opportunities to target components of the DNA damage response signaling network and the DNA repair pathways that are activated by radiotherapy to increase tumor radiosensitivity yet protect normal (healthy) cells. The authors discuss the influence of many biological and environmental factors that affect tumor and normal cell response to X-ray, proton, and carbon ion radiotherapy. Moreover, they examine the signaling pathways activated by radiotherapy and where opportunities are currently taken to sensitize tumor cells to radiotherapy. In continuation of the cellular response to radiation, they focus on repairing radiation-induced double-strand DNA breaks by non-homologous end-joining and homologous recombination. Furthermore, they discuss future avenues of promising drug combinations and potential unacceptable damage to healthy tissue. This review provides an excellent overview of the rapid development of compounds that target and sensitize tumor responses to tumors to “localized” radiotherapy in combination with “systemic” chemotherapeutics and the potential negative effect on healthy tissues.

The next article, by Gutierrez and O’Connor^[11]^, reports on DNA direct reversal repair and alkylating agent drug resistance. DNA direct reversal repair (DRR) is a unique repair mechanism that does not require a DNA synthesis step to “repair” the damage. Humans exhibit two different DRR pathways, the O6-methylguanine-DNA methyltransferase (MGMT) and the alkylated DNA repair protein B (AlkB) homologs. Many chemotherapeutic regimens contain an alkylating agent to which tumors acquire resistance that limits the use of this and other alkylating agents. This review highlights the mechanism of action of the DRR pathway enzymes, the development of drug resistance and discusses potential avenues to overcome resistance to alkylating agents. As alkylating agents are part of many chemotherapy-based treatment combinations, it is interesting to note that the induced damage is independent of the nucleotide, yet the level of damage to individual bases does not always correlate with therapeutic outcomes. This overview discusses the mechanistic differences between mono- and bi-functional agents, including the different alkylating adducts they form. Moreover, they review the simplest form of error-free DNA repair, DRR, the enzymes involved, and their catalytic mechanism, the alkylated bases they reverse, and the dependency on the mismatch repair pathway for successful treatment outcomes with alkylating agents. This review also highlights the mechanism of resistance cells develop to alkylating agents, including enzyme upregulation, changes in collaborative DNA repair pathways, and glutamine metabolism. Importantly, strategies to exploit these characteristics are discussed to promote successful therapeutic outcomes.

Saliba *et al.*^[12]^ take a mechanistic angle in their overview on the continuing occurrence of treatment-induced drug resistance in acute myeloid leukemia (AML). Despite successes and improvements in combination treatment of primary and secondary AML, about half the responders still show relapse after 18 months. Acquired drug resistance is still a significant roadblock in achieving a prolonged duration of response. They specifically focus on the current combination treatment of venetoclax with the hypomethylating agents (HMA) decitabine or azacytidine that improves outcomes in older patients over the standard of care therapies. HMAs are pyrimidine analogs that are incorporated into the DNA and inhibit the action of DNA methyltransferases (DNMT). They discuss the change in treatment with high cytotoxic doses to long exposures with a lower dose that affects epigenetic modifications via DNA hypomethylation that stimulates differentiation and tumor suppression without cell cycle arrest. They reviewed the differences in the mechanism of action of the different hypomethylating pyrimidine analogs and the cellular response they evoke, including those that cause drug resistance. In addition, they review the mechanism of action of venetoclax, an oral inhibitor of the anti-apoptotic protein BCL2, which, together with BCLX_L_ and MCL1, are frequently overexpressed in AML. Several potential mechanisms of acquired venetoclax resistance in AML and other leukemias are discussed, including modulation of expression levels of the BCL2 target and compensatory upregulation of BCL2 paralogs and acquired mutations in BCL2 that affect drug binding. Moreover, the mechanism of action and the development of resistance to this combination therapy are discussed from a molecular viewpoint. They conclude that this combination therapy is successful, but it is critical to evaluate modification in dosing schedules to avoid acquired resistance.

Recent successes with PARP inhibitors have led to investigations of other DNA repair targets as potential treatments. One such target is CHK1, which is an important regulator of the DNA repair checkpoint activated upon replication stress. Inhibition of CHK1 results in increased replication stress, accumulation of unrepaired DNA double-strand breaks, and cell death through apoptosis and mitotic catastrophe. Several CHK1 inhibitors have shown preclinical and clinical activity, and as with most targeted therapies, acquired resistance is an issue, including compensatory PI3K and MAPK pathway activation and induction of anti-apoptotic proteins. In their report, Lee *et al.*^[13]^ hypothesized that the upstream EGFR pathway could also serve as a resistance mechanism and that inhibition of EGFR would enhance the anti-tumor activity of the CHK1 inhibitor prexasertib in triple-negative breast cancer. Indeed, EGFR activation with EGF reduced cellular sensitivity to CHK1 inhibition, and conversely, inhibition of EGFR with erlotinib enhanced cellular cytotoxicity with prexasertib in several *in vitro* and *in vivo* models of triple negative breast cancer. These data are consistent with similar EGFR/CHK1 combinations in other tumor types, including head and neck cancers^[14]^. A recently reported clinical trial demonstrated that prexasertib could be safely combined with cetuximab and radiation^[15]^. However, biomarkers to select the patients most likely to benefit from these combinations are still needed.

It is well-established that genomic instability is a hallmark of carcinogenesis, and a mutation in *TP53* is an initiating event that triggers downstream activation of oncogenic pathways leading to cancer. Interestingly, Dr. Juhlin^[16]^ discusses aberrant DNA repair as a process that may facilitate clonal evolution of well-differentiated thyroid carcinoma into anaplastic thyroid cancer. With recent advances in next-generation sequencing technology, there is an increased ability to interrogate the genomic landscape of thyroid cancers and track phylogenetic clusters as tumors progressively de-differentiated into anaplastic thyroid cancer. Importantly, mismatch repair deficiency was noted, and the potential benefit of immune checkpoint inhibitors in this setting must be recognized for this aggressive disease.

Another mechanism by which DNA repair influences cancer treatment is by affecting tumor response to radiation. Fabbrizi and Parsons^[17]^ report on current and future perspectives of DNA damage response to enhance radiation for head and neck cancer. Authors discuss the differential radiation sensitivities of HPV-associated *vs.* non-HPV-associated head and neck cancer, likely due to ineffective DNA damage checkpoints and DNA repair mechanisms in HPV-associated head and neck cancers. They also discuss the role of tumor hypoxia in radiation resistance. Based on these pathways, radiation sensitization strategies are presented, including inhibitors of DNA repair proteins, such as PARP, ATR, and DNA-PK, as well as cell cycle checkpoints CHK1 and WEE1. The role of particle therapy (protons, carbon ions) in overcoming radiation resistance is also discussed. As radiation is one of the main modalities of treatment for head and neck cancer, there is a clear need to identify effective combinatorial strategies that can be tested in future clinical trials.

In summary, more work is needed to advance the field regarding rationally combining therapies targeting DNA repair with other agents, including immunotherapy. The best stimulus to further enhance the targeting of DNA repair is the realization that cancer cells have a greater dependency on DNA damage response and DNA repair than normal cells. Recent evidence also points to an interaction between DNA damage and the immune system^[18-20]^, and multiple trials are ongoing that are investigating combinations of DNA damage response targeting agents with immune checkpoint inhibitors. While these factors illustrate the promise of targeting DNA damage response, a clinical challenge has been to ascertain for each patient whether the cancer will have a long-term response to these agents. There is a dire need to uncover additional biomarkers that predict response/resistance, including detection tools to identify a DNA repair “fingerprint” as well as other oncogenic driver pathways in order to select the optimal therapeutic combinations and to identify early relapse of the disease that may inform novel interventions to combat resistance.
